# Analysing complex excitation patterns in cardiac tissue using wave event networks

**DOI:** 10.3389/fnetp.2025.1674919

**Published:** 2025-11-18

**Authors:** Hans Friedrich Von Koeller, Alexander Schlemmer, Stefan Luther, Yannic Döring, Niels Voigt, Ulrich Parlitz

**Affiliations:** 1 Research Group Biomedical Physics, Max Planck Institute for Dynamics and Self-Organization, Göttingen, Germany; 2 Institute for the Dynamics of Complex Systems, University of Göttingen, Göttingen, Germany; 3 Institute of Pharmacology and Toxicology, University Medical Center Göttingen, Göttingen, Germany; 4 German Center for Cardiovascular Research (DZHK), Partner-Site Lower-Saxony, Göttingen, Germany; 5 Cluster of Excellence “Multiscale Bioimaging: From Molecular Machines to Networks of Excitable Cells” (MBExC), University of Göttingen, Göttingen, Germany

**Keywords:** wave tracking, cardiac dynamics, network physiology, cardiac arrhythmias, atrial and ventricular fibrillation, polymorphictachycardia, spontaneous emissions, focal activity

## Abstract

Cardiac dynamics is governed by complex electrical wave patterns, with disruptions leading to pathological conditions like atrial or ventricular fibrillation. Experimentally electrical excitation waves can be made visible by optical mapping using fluorescent dyes. While this imaging technique has enabled detailed studies of cardiac wave dynamics, the manual analysis of activation and phase maps often limits the ability to systematically identify and quantify wave patterns. This study employs a wave tracking algorithm that constructs a graph-based representation of wave dynamics. With that the algorithm detects key events such as wave emergence, splitting, and merging. Applied to both simulated cardiac tissue and experimental data from cell cultures, the algorithm identifies and quantifies wave patterns as *wave event networks*. Initial results demonstrate its utility in filtering for and focusing on dominant dynamics, providing a robust tool for analyzing cardiac wave patterns. This approach offers potential applications, e.g., to study the effects of external stimuli on cardiac excitation patterns and to better understand the mechanisms involved.

## 1 Introduction

Cardiovascular diseases, including atrial and ventricular fibrillation, exact a significant death toll worldwide, underscoring the critical need for accurate methods to track and analyse cardiac dynamics Gray et al. (1998). The heart’s rhythmic contractions are governed by regular electrical waves, orchestrating its synchronized motion. However, disturbances in this harmony can lead to the formation of spiral waves or scroll waves in the cardiomyocard, eventually culminating in fibrillation. Detecting and monitoring these aberrant waves is a pivotal step towards understanding the mechanisms behind this condition and developing effective interventions Luther et al. (2011); Lilienkamp and Parlitz (2020); Rappel et al. (2022); Lilienkamp et al. (2022); Buran et al. (2023); Steyer et al. (2023); Suth et al. (2024); Garzón and Grigoriev (2024); Aron et al. (2025).

The complexity of wave patterns in cardiomyocyte monolayers observed through optical voltage mapping is often quantified by manually analyzing datasets (Maizels et al., 2024). The computation of activation or phase maps can aid this process, making it easier to identify rotors for example, by locating phase singularities. These can then be quantified and tracked over time (Iyer and Gray, 2001; Kiseleva et al., 2024; Portero et al., 2024; Bingen et al., 2014). However, this approach cannot be applied to wave patterns that do not include phase singularities, such as leading-circle reentries around fibrotic cores. Alternatively, a line scan analysis can allow researchers to monitor complex activity over time. However, it only takes into consideration the activity along a line, ignoring the rest of the two-dimensional recording (Feola et al., 2017; Bingen et al., 2013). Recently, progress has been made in the automatic detection of specific wave patterns, e.g., reentry circuits, through the construction of directional graphs that describe the directions of wave propagation using cross-correlation analyses Bezerra et al. (2025).

In this article, we present and evaluate a wave tracking algorithm for analyzing cardiac dynamics. The concept is similar to the method presented in Rogers (2004) where tracking wave fronts is used in combination with phase singularity analysis. The approach we describe in this article offers a different perspective on tracking cardiac dynamics than phase singularity analysis: it binarizes the tissue into active and inactive zones and classifies connected parts as waves. By comparing consecutive points in time one can obtain low-dimensional meta data like location and time of emerging waves or numbers and locations of mergers and splits of interacting waves.

We apply this algorithm to simulations of cardiac tissue, generated using the Fenton-Karma model Fenton et al. (2002). Furthermore, we use optical mapping data of cardiac monolayers to evaluate the application of the algorithm to experimental data. The algorithm is expected to offer particular advantages when the heart tissue exhibits high focal activity, such as *spontaneous calcium releases* Voigt et al. (2012). Another possible application of this method is to automatically quantify how agents that modulate sarcoplasmic-reticulum Ca^2+^ release (e.g., high-dose caffeine) alter the incidence and spatiotemporal organization of such spontaneous events.

## 2 Methods

In this section the concept and implementation of the wave tracking algorithm will be introduced. Furthermore, we describe the simulations that were used to generate test data.

### 2.1 Wave tracking algorithm

**FIGURE 1:**
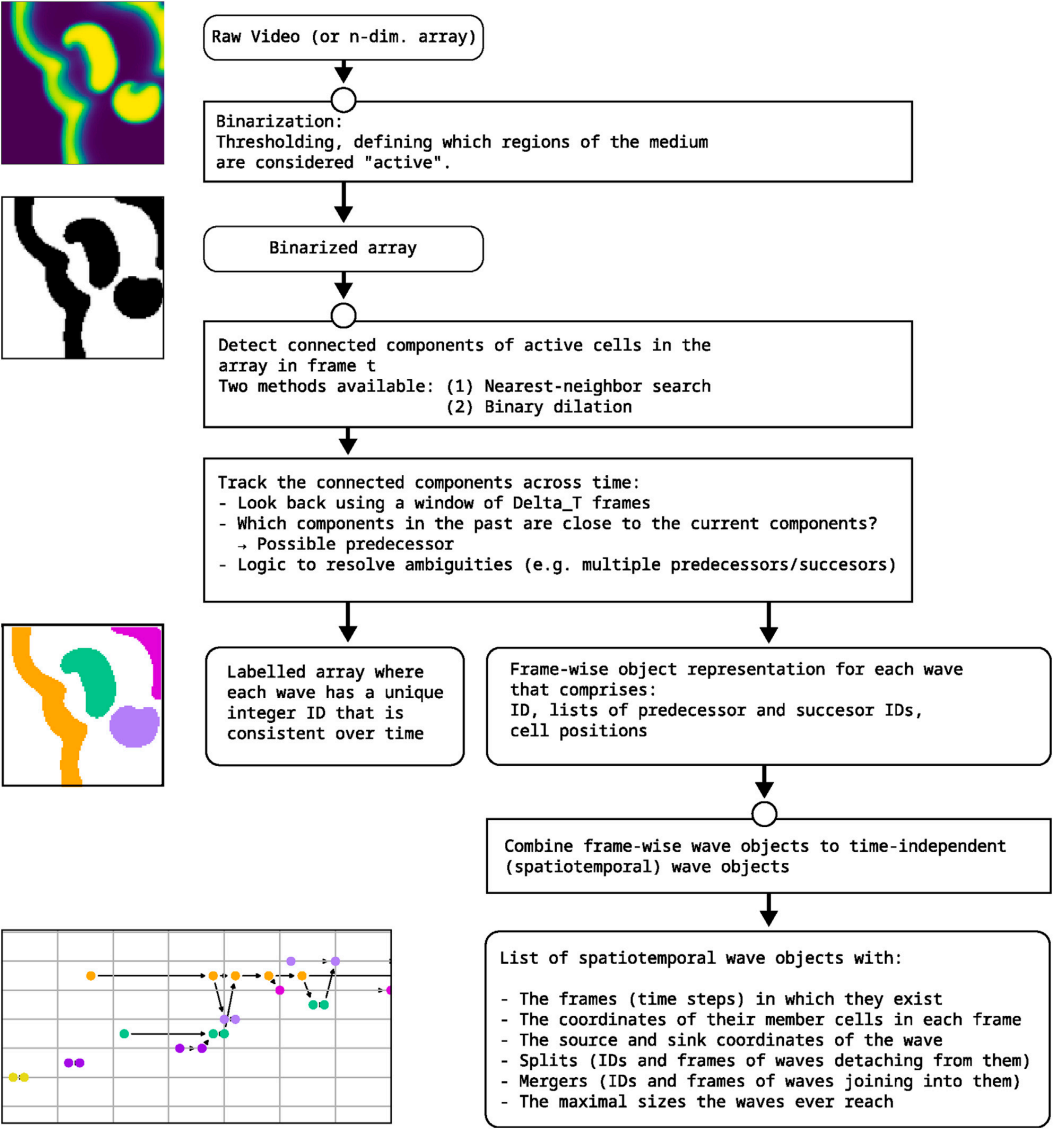
Schematic overview over the algorithm for generating wave event networks. Further details of its implementation can be found in the code Schlemmer (2025).

We will refer to our approach to describing and tracking cardiac dynamics as the “wave tracking algorithm”. Figure 1 shows a flow-chart of the main processing steps. The algorithm can essentially be devided into three distinct parts:

- Binarization (Thresholding): Connected parts of the tissue where the membrane potential over a certain threshold is defined as a wave and identified by the algorithm.
- Frame-wise detection of waves: Frame-wise wave objects are created for each frame by analysing the relation between connected components in successive frames.
- Spatiotemporal wave objects: The frame-wise wave objects are connected to spatiotemporal wave objects by analysing their interactions.

The spatiotemporal wave objects created in the last step of the algorithm provide access to time-independent information about the waves which are relevant for subsequent analysis:

- Time and location of wave emergence
- Development of the wave size over time
- Times and locations of interactions with other waves: Splits and mergers
- Time and location of the disappearance of the wave

In the following subsections the algorithm is described more in detail.

#### 2.1.1 Spatial tracking

**FIGURE 2:**
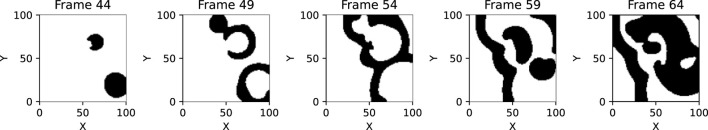
This figure shows the binarized wave parts in five exemplary frames of a Fenton-Karma simulation with spontaneous excitations every five frames and with wave pixels (pixels above a certain threshold, 0.4 in this case) being shown in black and non-wave pixels being shown in white.

To differentiate in the raw data between wave and non-wave pixels (binarize the data, differentiating between excited wave pixels and non-wave pixels) a threshold is applied to the variable corresponding to the voltage. Theoretically, there exists always a well-defined threshold of excitation of the excitable medium. In practice, the determination of the threshold may not always be a straight-forward task as it may depend on the properties of the excitable medium and the experimental conditions (e.g., sensor noise, exposure times, etc.). However, in the considered cases a reasonable threshold that separates excited pixels from non-excited pixels could easily be found by hand. For more complex scenarios and experimental data the threshold has to be chosen more carefully. Other methods to empirically determine the threshold include e.g., Otsu’s algorithm Otsu (1979) and minimizing Gini impurity Breiman et al. (1984). An example for binarization can be seen in Figure 2.

In experimental settings the parameter value of the threshold can have a huge impact on the outcome of the wave-tracking procedure: Low values typically lead to larger connected areas and therefore to a lower number of waves. Setting too low values for the threshold will therefore not lead to meaningful information as the algorithm will be unable to detect wave interactions, but only detect a single big wave. In difficult situations the threshold parameter can be determined using a parameter scan. By comparing the output of the algorithm with a manually determined number of waves the parameter can be fine-tuned.

**FIGURE 3:**
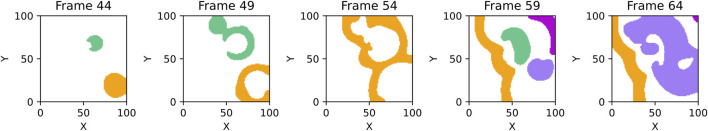
This figure shows connected wave parts in different colors for the same simulation as in Figure 2. As waves are tracked throughout the simulation, their colors stay constant over time.

Within the binarized data connected parts are labeled either with nearest neighbor search or with one or more iterations of binary dilation in combination with a labelling of connected components. This behavior can be seen in every frame of Figure 3. As especially for noisy or experimental data strict connectedness might not be a suitable criterion it is possible to set a radius within which active pixels are still considered connected.

Using the information which pixels at which time constitute a connected part, wave objects are created and saved by the implemented wave tracking algorithm, for each wave one object, and filled with information about the location and size of the wave at one point in time. To be able to sensibly deal with high noise or otherwise impaired input data where wave parts may have a small gap between them, the implementation of the wave tracking algorithm allows for wave parts within a certain radius to be considered as one wave if there is a gap in the binarized data smaller than the set radius parameter.

#### 2.1.2 Temporal tracking

After the waves are spatially connected they need to be connected temporally. For this to be achieved, waves at time t (potential successors) and waves at time t−1 (potential predecessors) are considered.

For each wave at time t potential predecessors are identified using nearest neighbour search. Starting with the smallest wave it is checked whether there is exactly one predecessor candidate for this successor and one successor candidate for this predecessor. In this case it is easily determined that the incoming predecessor wave travels forth as the successor wave without any interactions with other waves.

If there is one predecessor candidate and there are multiple successor candidates for this potential predecessor the predecessor candidate of this wave will be removed temporarily from the list of predecessor candidates. The wave will be incorporated into a list of waves, that could not be directly classified. This order of solving the uncertainties in assignment is supposed to favor larger waves as those will be dealt with later on when the amount of waves with multiple successor candidates is lower due to them being removed temporarily. Later on this wave will be reconsidered and also potentially classified as a split of waves, if it turns out that both successors originate from the same predecessor.

If there are multiple predecessor candidates and only one successor for every one of them (that also is no successor candidate for any other wave) the waves are merged into that one successor.

In the case that there are multiple predecessor candidates and multiple successor candidates the wave will be reappended at the end of the list of waves at t and put into the list of not directly classifiable waves but only if it isn’t already in this list. Otherwise it will use just one of the possible predecessors as the predecessor.

If there are no predecessor candidates at all a new wave is created. This may however become the product of a split as the predecessor candidate may have been removed if there were multiple successors.

### 2.2 Datasets

#### 2.2.1 Fenton-Karma model simulations

The dataset that is referred to as the ‘simulated data’ within this paper was generated using the MediaSim tool Bittihn (2014) and the Fenton-Karma model with the set of parameters shown in Table 1 on a 100×100 grid with dx=dy=0.8 , a diffusion coefficient of D=0.15 and no-flux boundary conditions. The Fenton-Karma model, originally introduced in Fenton and Karma (1998), is a three variable model to simulate cardiac dynamics. It is used because of its straight forward set up of pacings in the action potential while still ensuring suitable cardiac-like behaviour of the voltage-like variable V that is used for further analysis in this paper.

**TABLE 1:** Parameters of the Fenton-Karma simulation based on set 8 from Fenton et al. (2002).

Parameter	Value	Parameter	Value	Parameter	Value
$C_{m}$	1	$\tau_{v}^{+}$	13.03	$\tau_{v1}^{-}$	19.6
$\tau_{v2}^{-}$	1,250	$\tau_{w}^{+}$	800	$\tau_{w}^{-}$	40
$g_{fi}$	2.222	$\tau_{r}$	33.25	$\tau_{si}$	29
k	10	$u_{\mathrm{csi}}$	0.85	$u_{c}$	0.13
$u_{v}$	0.04	$k_{2}$	500	$\tau_{0}$	12.5

The governing equations of the Fenton-Karma model can be written, following the nomenclature of Fenton et al. (2002), as an equation for the transmembrane potential V(x,y,t)∈[0,1] coupled to two gate variables v,w∈[0,1] :

$$\partial_{t}V=\nabla \;\cdot \;\left(D\;\nabla V\right)-\frac{I_{\text{fi}}\left(V,v\right)+I_{\text{so}}\left(V\right)+I_{\text{si}}\left(V,w\right)}{C_{m}}+I_{\text{stim}}\left(x,y,t\right),$$

together with the gate ODEs specified below.

The three currents in the Fenton-Karma model are

$$\begin{array}{cc} I_{\text{fi}}\left(V,v\right) & =-g_{fi}\;\;v\;S_{1}\left(V\right)\;\left(V-u_{c}\right)\;\left(1-V\right),\\ I_{\text{so}}\left(V\right) & =\frac{V\;\left(1-S_{1}\left(V\right)\right)}{\tau_{0}}+\frac{S_{1}\left(V\right)}{\tau_{r}},\\ I_{\text{si}}\left(V,w\right) & =-\frac{w\;S_{2}\left(V\right)}{\tau_{si}}. \end{array}$$

where

$$S_{1}\left(V\right)=H_{k}\left(V-u_{c}\right),\;S_{2}\left(V\right)=H_{k}\left(V-u_{\mathrm{csi}}\right),\;S_{3}\left(V\right)=H_{k_{2}}\left(V-u_{v}\right),$$

are given by a smooth approximation of the Heaviside function

$$H_{k}\left(\xi \right)=\frac{1}{2}\left(1+\tanh\left(k\;\xi \right)\right)$$

with parameters k and $k_{2}$ (Table 1) setting the steepness.

The v –gate uses a mixed recovery time constant

$$\tau_{v}^{-}\left(V\right)=\left(1-S_{3}\left(V\right)\right)\;\tau_{v1}^{-}+S_{3}\left(V\right)\;\tau_{v2}^{-}.$$

The gate ODEs are:

$$\dot{v}=\left(1-S_{1}\left(V\right)\right)\;\frac{1-v}{\tau_{v}^{-}\left(V\right)}-S_{1}\left(V\right)\;\frac{v}{\tau_{v}^{+}},\;\dot{w}=\left(1-S_{1}\left(V\right)\right)\;\frac{1-w}{\tau_{w}^{-}}-S_{1}\left(V\right)\;\frac{w}{\tau_{w}^{+}}.$$

The simulations used a finite-difference solver with explicit time stepping and a timestep of Δt=0.05 . Every 10th simulation time unit the variables were saved as a simulation frame.

To simulate the spontaneous emissions mentioned in the introduction, spikes of activity were paced at a random spot on the tissue every 5 frames. The turbulent behaviour starts around frame 49, as soon as one pacing happens to occur sufficiently close to the last one.

#### 2.2.2 Experimental data

To show the applicability of the algorithm for real data the wave tracking algorithm was also applied to experimental data. For this purpose human induced pluripotent stem cell derived atrial cardiomyocytes were generated using previously established protocols (Kleinsorge and Cyganek, 2020; Seibertz et al., 2023). Cardiomyocytes were then plated onto a glass coverslip with a diameter of 22 mm that was coated with Matrigel. The cells were cultured for 7 days, allowing them to form a confluent monolayer. To study action potential duration and conduction properties, the monolayer was incubated with the voltage-sensitive dye Di-4-Anepps (35 μ M) (D1199, Invitrogen) for 7 min at 37 °C. Imaging was performed using a complementary metal-oxide semiconductor (CMOS) camera (Micam Ultima 10 × 10 mm^2^ sensor, Brainvision/Sci-Media). The recordings were taken at a frame rate of 500 fps. During the experiment, the monolayer was maintained at 37 °C in a HEPES-buffered bath solution containing: 140 mM NaCl, 16 mM KCl, 1 mM MgCl2, 2 mM CaCl2, 10 mM HEPES, 10 mM glucose, pH 7.4. The monolayer was electrically point-stimulated close to the edge at the bottom right at a stimulation frequency of 2 Hz using a custom-built platinum/iridium bipolar electrode. The voltage indicator was excited with peak wavelengths of 531 nm using an LED (LEX3 illumination system, Sci-Media), and emission was passed through a 600 nm long-pass filter.

Making the raw input data usable for the algorithm requires a few key preprocessing steps:

First, to differentiate between pixels that resemble tissue and those that do not (as the monolayer sits in a circular form on the coverslip with an overlapping electrode, which creates borders in the recording that lack tissue), the variance of each pixel over the time series is calculated. By distinguishing between high-variance and low-variance pixels (in this case a clearly bimodal distribution), a mask of tissue pixels can be created.

**FIGURE 4:**
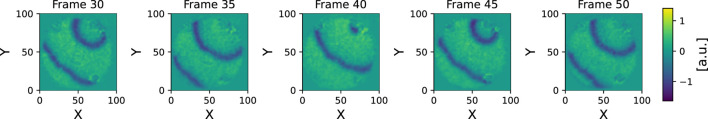
Here normalized and filtered experimental data is displayed for multiple frames. After these preprocessing steps the data is fed into the wave tracking algorithm.

The tissue data is then normalized by subtracting the mean and dividing by the standard deviation. Additionally, a small Gaussian filter is applied to reduce noise and improve runtime efficiency. In the displayed figures (e.g., in Figure 4), this filter has a kernel with a standard deviation of one frame taken into account by the wave tracking algorithm in the temporal dimension (encompassing multiple raw frames due to unnecessarily high temporal resolution of the raw data, a downsampling of 10 times was used after the preprocessing and before applying the wave tracking algorithm) and one pixel in both x and y directions. This filter helps optimize the algorithm’s performance by reducing the number of spatially and temporally close waves, which otherwise would negatively impact runtime. It also enhances algorithmic robustness, as a high local density of potential predecessors and successors could increase the likelihood of misattribution.

This preprocessing makes the data suitable for binarization and the subsequent wave tracking algorithm. In this case the radius parameter discussed in Section 2.1.1 was set to 3  px.

## 3 Results

**FIGURE 5:**
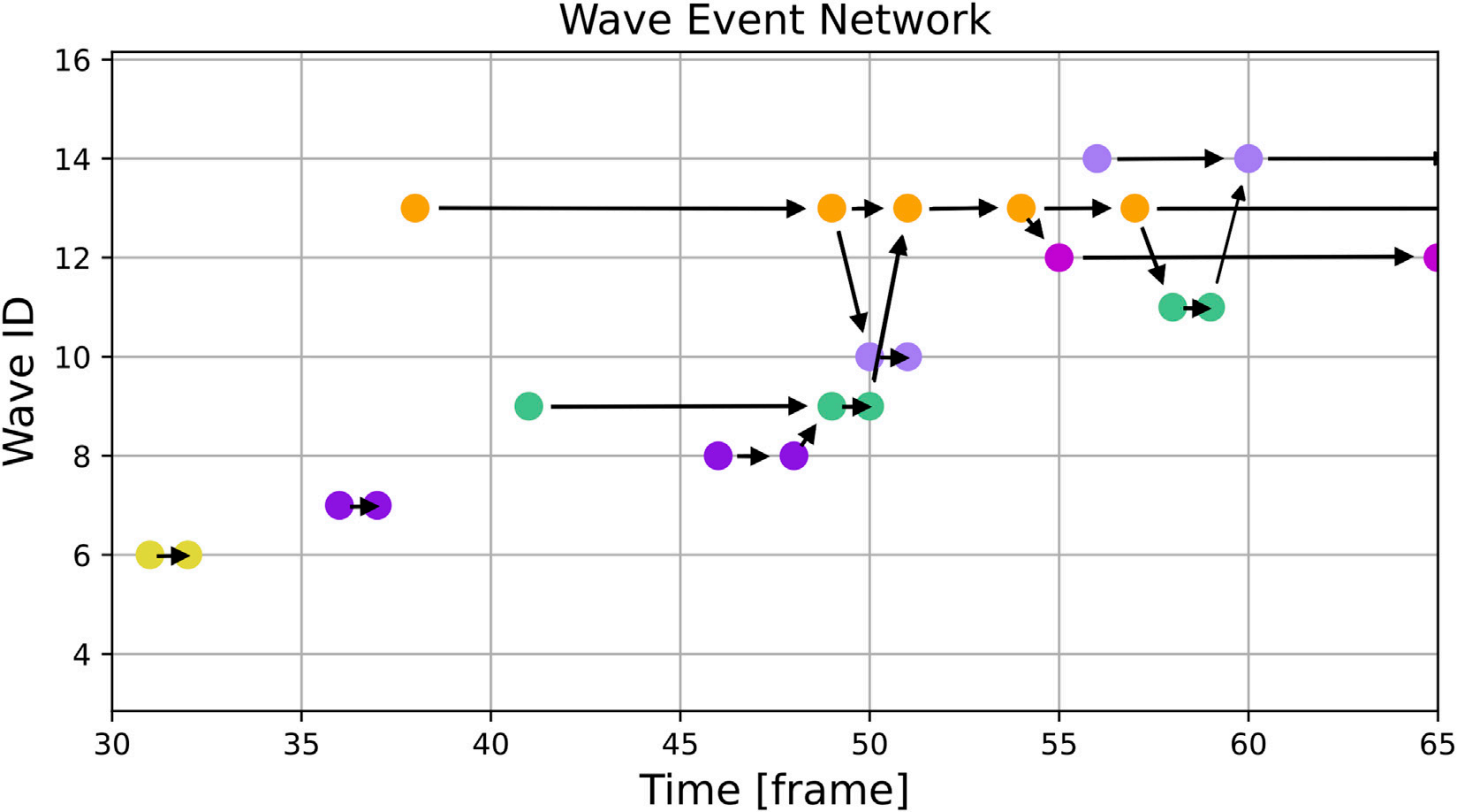
This figure shows an excerpt of the same simulation as Figure 3 but instead of single frames it displays the graph with the colored nodes corresponding to the colored waves. The horizontal arrows show continuing waves and the diagonal ones show splits and mergers of waves. One can see, for example, the regular pacings without any interactions with other waves in frames 31 and 36 and the turquoise wave (ID 9) merging into the orange one (ID 13) on frame 51. Additionally, one can see, that due to the evolution of the wave patterns and the threshold used, the violet wave (ID 7) is not recognized as the source of the orange wave (ID 13).

We applied the algorithm to the simulated (see Section 2.2.1) dataset and to the experimental dataset (Section. 2.2.2). For getting an overview of the waves detected by the procedure we show for each dataset a special visualization that we refer to as the “Wave Event Network”. An example can be seen in Figure 5. The horizontal axis of this plot displays the time in frames measured since the start of the recording. On the vertical axis the “Wave ID”, which is assigned to individual waves by the algorithm, is shown. For better visual identification, the visualization method assigns a random color to each “Wave ID”. Each filled circle in the plot corresponds to a distinct wave event which can be one of the following:

- wave creation
- wave annihilation
- wave merger
- wave split

Wave events are connected by black arrows. If two waves merge, there is a wave event displayed for the last frame that contains the merged wave and another wave event in the next frame where both waves are combined into a single one. An arrow is drawn indicating the direction of the merger. Similarly, a split is indicated by two events and an arrow pointing to the event of the newly created wave.

Videos showing the full scenarios used within this paper and the respective representation using the wave tracking algorithm can be found in the supplementary materials.

### 3.1 Simulated data

The results of the wave tracking algorithm applied to simulated data can be seen in Figures 3, 5. Figure 3 shows the spatial distribution of waves with their wave ID indicated by a color. Figure 5 shows the corresponding wave event network with the same color code.

By comparing both figures, the interpretation of the wave event network becomes clearer: E.g., in frame 59 four distinct waves can be spotted:

- ID 11: green color
- ID 12: purple color
- ID 13: orange color
- ID 14: lilac color

Wave ID 11 is about to be merged with wave ID 14 in frame 60. The result can be seen in Figure 3 where only three of the waves remain.

A similar transition can be observed from frames 49 to 54: Several minor events fall into this interval, but the effective transition is a merger of two bigger waves (wave IDs 9 and 13).

### 3.2 Experimental data

**FIGURE 6:**
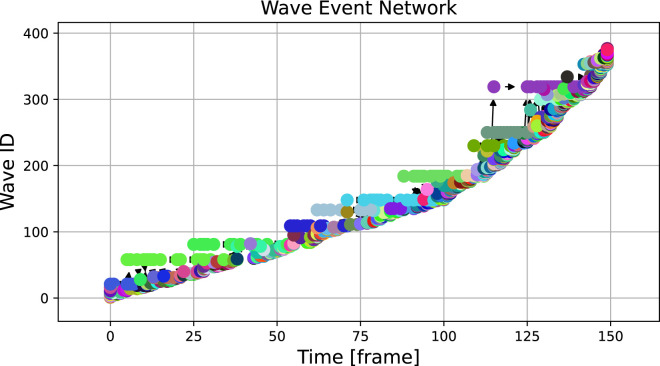
The graph over 150 frames of the experimental video, binarized at a threshold of 0.2, shows a large number of waves with very short lifetimes. The analysed excerpt was captured at 500 frames per second and downsampled 10 times, hence the 150 frames correspond to 3 s of real time.

When applied to the experimental dataset, the wave tracking algorithm provides the result shown in Figure 6. Noise and heterogeneities inherent to experimental data lead to a much higher number of waves and a cluttered visualization. The plot indicates that the lifetime of detected waves is on average much shorter than in the numerical case and broader structures are difficult to identify by eye.

**FIGURE 7:**
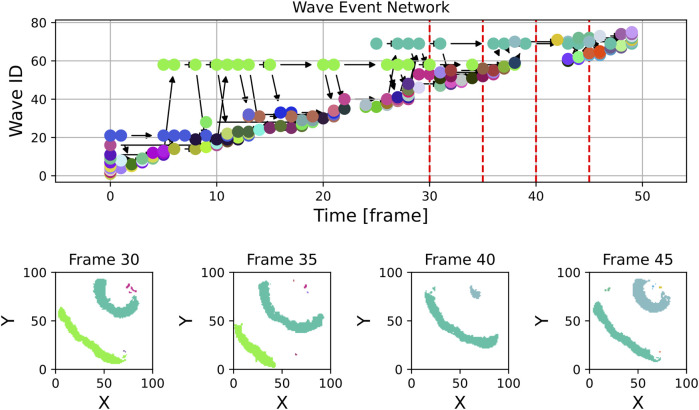
This figure shows an unfiltered excerpt from the experimental graph along with a few example frames from the experimental data. While some of the many waves visible in the frames and at the wave event network may grow into significant waves on the tissue, others are likely artifacts caused by noise or other unwanted effects at the border.

Zooming into the plot, which was done in Figure 7, reveals more structure: In addition to the high number of small waves, some structures that are stable over a longer period of time become visible in the light green (wave ID 58) and teal (wave ID 68) wave event markers. In Figure 7, some panels displaying spatial snapshots of the data were extracted from frames 30, 35, 40 and 45 (also highlighted with dashed red lines in the upper panel). Here, indeed, it can be seen that there are at any shown time step one or two dominant waves that are accompanied by tiny speckle patterns, especially at the Petri dish borders.

**FIGURE 8:**
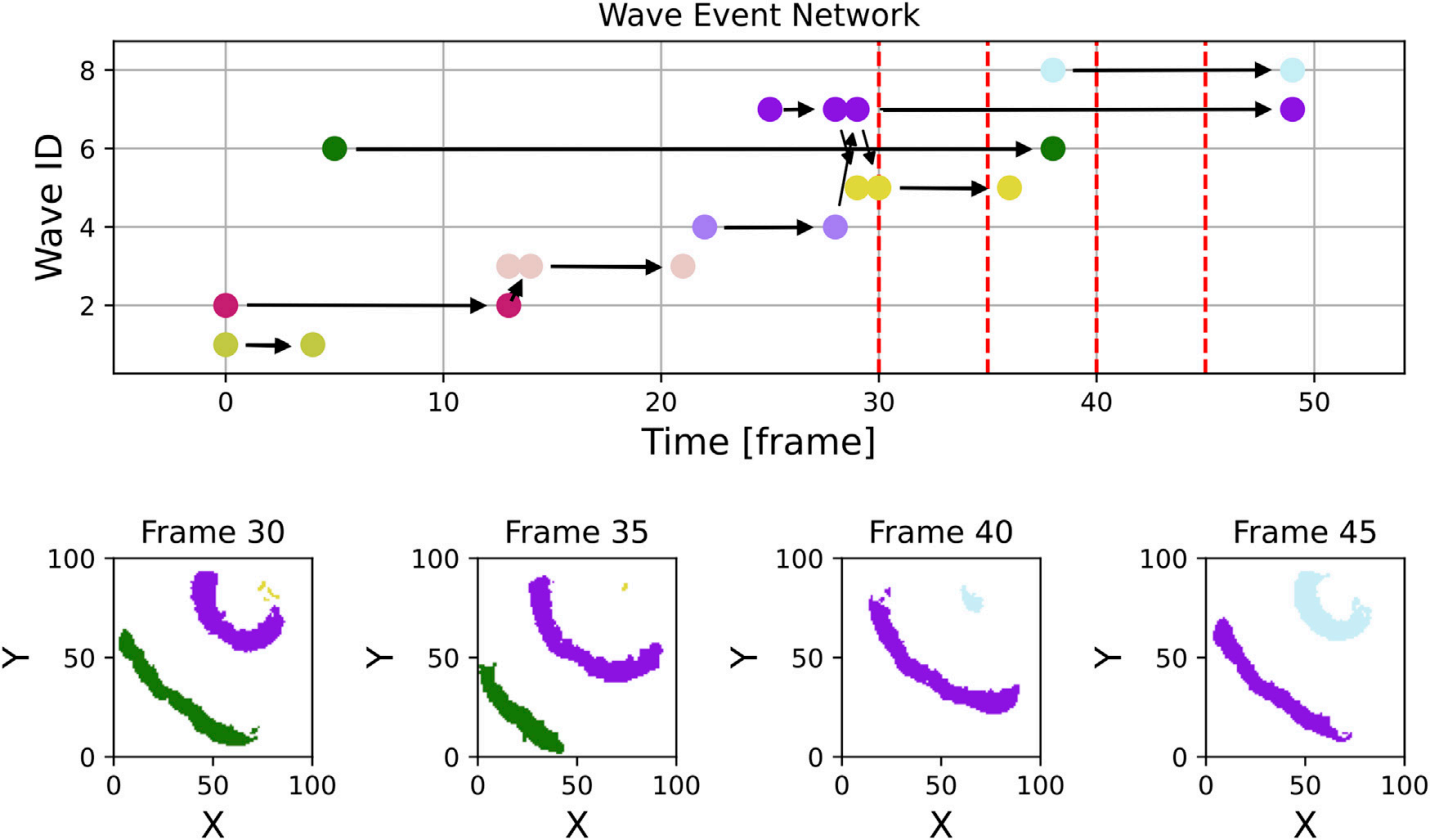
In contrast to Figure 7, this figure displays the section of experimental data, but with waves filtered to exclude those with a lifetime of only one frame and a maximum size of fewer than 20 pixels. Most small artifacts have been effectively removed, the wave event network now contains essentially only waves showing the relevant dynamics. The violet wave in the top right corner of frame 30 is considered as one wave object due to the used radius parameter of 3  px.

**FIGURE 9:**
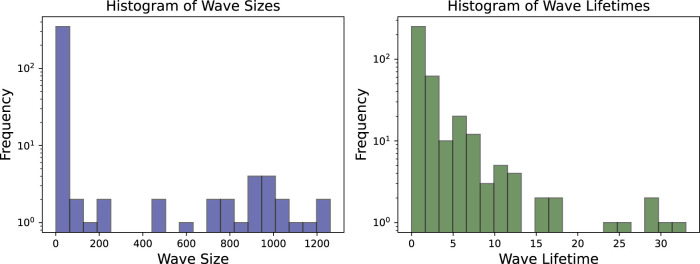
Both wave sizes and wave lifetimes for the experimental video are displayed at a logarithmic scale. Especially for the wave sizes it is clear, that a large number of very small waves are observed which are in many cases insignificant for the actual dynamics.

By filtering out waves with a lifetime of one frame or less and a maximum size of fewer than 20 pixels, as shown in Figure 8, the number of wave objects considered reduces from nearly 400 to fewer than 50. The remaining waves more accurately represent the dominant dynamics on the tissue. The histogram of wave sizes and wave lifetimes for this video is displayed in Figure 9 and justifies filtering for small wave sizes and lifetimes.

### 3.3 Detection errors

There are several error types which may cause differences between expected and actual results.

One error type is related to the thresholding step: if there is activity spreading with a peak excitation lower than the threshold this excitation will not be considered as a wave during binarization. A similar problem appears if there are spurious holes within one wave that split the wave into multiple.

Another possible error is the misattribution of predecessors or successors. Especially if there are many different candidates and a comparatively coarse frame rate the heuristics in the implementation of the algorithm (e.g., assigning largest waves first) might break down.

The last error to be discussed here is perceived behaviour at the edge of the domain. One example of that is shown in Figure 5, where between frame 54 and 55 a wave splits up. Looking at Figure 3 it is clear, that there is no actual split of the wave, instead the wave hits the boundary of the domain.

## 4 Conclusion

In this article we presented an algorithm that is able to track wave dynamics in cardiac tissue and represent it as a wave event network. The algorithm is capable of automatically detecting wave structures in numerical and experimental datasets observed in excitable media. The method is straight-forward and closely resembles manual inspection of wave-structures which is employed in many scientific studies Maizels et al. (2024); Óscar et al. (2021); Askar et al. (2012); Harlaar et al. (2022); Bingen et al. (2015) and which might be inefficient and not systematically reproducible Wilson et al. (2014). The method, we present here, generates overview of the wave dynamics which can be used to guide interpretation. The unique advantages include the independence from phase singularities and the ability to gather high-level wave features and process them in a graph. Furthermore, this graph can by used in different ways to deal with numerical noise and other artifacts. Furthermore, it is possible to extract features from the computed wave objects (like wave lifetimes, locations and time periods of wave events, wave sizes) that can be the input to subsequent data analysis procedures Datseris and Zelko (2024). This will allow to make use of wave properties in studies comparing different datasets of excitable media in large quantities efficiently.

One special feature of the algorithm is that it can be used to filter data using abstract properties (like the size of waves or the lifetime) as it was demonstrated for the experimental dataset. In contrast to image processing methods like kernel smoothing, this approach is much more specific to the properties of the excitable system and might be able to achieve a higher precision when looking for important characteristics of the medium.

As for any other method, this procedure includes some parameters that can highly influence the results. Apart from a possible pre-processing of the image data (e.g., kernel smoothing) the most relevant parameter is the threshold of the binarization procedure. We found that by plotting the histogram of the image data, it is in most cases possible to identify a broader, stable range of threshold values that lead to a good separation between non-excited and excited areas of the tissue. Another possibility is to run the full algorithm on a smaller sample of the data set and tune the threshold parameter to a value that leads to the expected number of waves.

In general, we observed that the algorithm is very robust regarding the pre-processing of the image data and can, in principle, also be applied to unfiltered data.

## 5 Outlook

The approach presented here was designed with a focus on a quantitative investigation of wave properties. In this study, however, only a limited number of quantities that can in principle be extracted from the procedure have been investigated. As the algorithm provides access to many quantities, like the positions of specific wave events and time-resolved numbers of individual wave sizes and shapes, many possibilities exist for further automatic processing. These applications include supervised or unsupervised machine learning applications or statistical comparisons of different biomedical conditions studied in specific experiments. The tool can thus be considered a complement to prevalent methods for analyzing cardiac dynamics, like phase singularity analysis, dominant frequency maps or activation maps.

## Data Availability

The original contributions presented in the study are included in the article/supplementary material, further inquiries can be directed to the corresponding author.

## References

[B1] Aron M. Luther S. Parlitz U. (2025). Success rates of simulated multi-pulse defibrillation protocols are sensitive to application timing with individual, protocol-specific optimal timings. Front. Netw. Physiology 5, 1572834. 10.3389/fnetp.2025.1572834 40496437 PMC12148900

[B2] Askar S. F. Bingen B. O. Schalij M. J. Swildens J. Atsma D. E. Schutte C. I. (2012). Similar arrhythmicity in hypertrophic and fibrotic cardiac cultures caused by distinct substrate-specific mechanisms. Cardiovasc. Res. 97, 171–181. 10.1093/cvr/cvs290 22977008

[B3] Bezerra A. S. Hendrickx S. Van den Abeele R. Wülfers E. M. Verstraeten B. Lootens S. (2025). Cross-correlation as an alternative for local activation times for the analysis of reentries in directed graph mapping. Biomed. Signal Process. Control 106, 107716. 10.1016/j.bspc.2025.107716

[B4] Bingen B. O. Askar S. F. Schalij M. J. de Vries A. A. Pijnappels D. A. (2013). Prolongation of minimal action potential duration in sustained fibrillation decreases complexity by transient destabilization: reply. Cardiovasc. Res. 98, 156–157. 10.1093/cvr/cvt027 23396603

[B5] Bingen B. O. Engels M. C. Schalij M. J. Jangsangthong W. Neshati Z. Feola I. (2014). Light-induced termination of spiral wave arrhythmias by optogenetic engineering of atrial cardiomyocytes. Cardiovasc. Res. 104, 194–205. 10.1093/cvr/cvu179 25082848

[B6] Bingen B. O. Askar S. F. A. Neshati Z. Feola I. Panfilov A. V. De Vries A. A. F. (2015). Constitutively active acetylcholine-dependent potassium current increases atrial defibrillation threshold by favoring post-shock Re-Initiation. Sci. Rep. 5, 15187. 10.1038/srep15187 26487066 PMC4613729

[B7] Bittihn P. (2014). Complex structure and dynamics of the heart. Springer. 10.1007/978-3-319-12232-8

[B8] Breiman L. Friedman J. H. Olshen R. A. Stone C. J. (1984). Classification and regression trees. 1st edn. Chapman and Hall/CRC. 10.1201/9781315139470

[B9] [Dataset] Buran P. Niedermayer T. Bär M. (2023). Mechanism of defibrillation of cardiac tissue by periodic low-energy pacing. 10.1101/2023.03.16.533010

[B10] Datseris G. Zelko J. S. (2024). Physiological signal analysis and open science using the Julia language and associated software. Front. Netw. Physiology 4, 1478280. 10.3389/fnetp.2024.1478280 39569020 PMC11577965

[B11] Fenton F. Karma A. (1998). Vortex dynamics in three-dimensional continuous myocardium with fiber rotation: filament instability and fibrillation. Chaos 8, 20–47. 10.1063/1.166311 12779708

[B12] Fenton F. H. Cherry E. M. Hastings H. M. Evans S. J. (2002). Multiple mechanisms of spiral wave breakup in a model of cardiac electrical activity. Chaos 12, 852–892. 10.1063/1.1504242 12779613

[B13] Feola I. Volkers L. Majumder R. Teplenin A. Schalij M. J. Panfilov A. V. (2017). Localized optogenetic targeting of rotors in atrial cardiomyocyte monolayers. Circulation Arrhythmia Electrophysiol. 10, e005591. 10.1161/CIRCEP.117.005591 29097406

[B14] Garzón A. Grigoriev R. O. (2024). Ultra-low-energy defibrillation through adjoint optimization. Chaos An Interdiscip. J. Nonlinear Sci. 34, 113110. 10.1063/5.0222247 39496221

[B15] Gray R. A. Pertsov A. M. Jalife J. (1998). Spatial and temporal organization during cardiac fibrillation. Nature 392, 75–78. 10.1038/32164 9510249

[B16] Harlaar N. Dekker S. O. Zhang J. Snabel R. R. Veldkamp M. W. Verkerk A. O. (2022). Conditional immortalization of human atrial myocytes for the generation of *in vitro* models of atrial fibrillation. Nat. Biomed. Eng. 6, 389–402. 10.1038/s41551-021-00827-5 34992271

[B17] Iyer A. N. Gray R. A. (2001). An experimentalist’s approach to accurate localization of phase singularities during reentry. Ann. Biomed. Eng. 29, 47–59. 10.1114/1.1335538 11219507

[B18] Kiseleva D. G. Dzhabrailov V. D. Aitova A. A. Turchaninova E. A. Tsvelaya V. A. Kazakova M. A. (2024). Arrhythmogenic potential of myocardial edema: the interstitial osmolality induces spiral waves and multiple excitation wavelets. Biomedicines 12, 1770. 10.3390/biomedicines12081770 39200234 PMC11351629

[B19] Kleinsorge M. Cyganek L. (2020). Subtype-directed differentiation of human ipscs into atrial and ventricular cardiomyocytes. Star. Protoc. 1, 100026. 10.1016/j.xpro.2020.100026 33111079 PMC7580117

[B20] Lilienkamp T. Parlitz U. (2020). Terminating transient chaos in spatially extended systems. Chaos An Interdiscip. J. Nonlinear Sci. 30, 051108. 10.1063/5.0011506 32491910

[B21] Lilienkamp T. Parlitz U. Luther S. (2022). Taming cardiac arrhythmias: terminating spiral wave chaos by adaptive deceleration pacing. Chaos An Interdiscip. J. Nonlinear Sci. 32, 121105. 10.1063/5.0126682 36587312

[B22] Luther S. Fenton F. H. Kornreich B. G. Squires A. Bittihn P. Hornung D. (2011). Low-energy control of electrical turbulence in the heart. Nature 475, 235–239. 10.1038/nature10216 21753855 PMC3153959

[B23] Maizels L. Heller E. Landesberg M. Glatstein S. Huber I. Arbel G. (2024). Utilizing human-induced pluripotent stem cells to study cardiac electroporation pulsed-field ablation. Circulation Arrhythmia Electrophysiol. 17, e012278. 10.1161/CIRCEP.123.012278 38344845 PMC10949974

[B24] Óscar S.-M. Ramirez R. J. Takemoto Y. Ennis S. R. Garcia-Iglesias D. Wang S. (2021). Panoramic endocardial optical mapping demonstrates serial rotors acceleration and increasing complexity of activity during onset of cholinergic atrial fibrillation. J. Am. Heart Assoc. 10, e022300. 10.1161/JAHA.121.022300 34726079 PMC8751940

[B25] Otsu N. (1979). A threshold selection method from gray-level histograms. IEEE Trans. Syst. Man, Cybern. 9, 62–66. 10.1109/TSMC.1979.4310076

[B26] Portero V. Deng S. Boink G. Zhang G. de Vries A. Pijnappels D. (2024). Optoelectronic control of cardiac rhythm: toward shock-free ambulatory cardioversion of atrial fibrillation. J. Intern. Med. 295, 126–145. 10.1111/joim.13744 37964404

[B27] Rappel W.-J. Krummen D. E. Baykaner T. Zaman J. Donsky A. Swarup V. (2022). Stochastic termination of spiral wave dynamics in cardiac tissue. Front. Netw. Physiology 2, 809532. 10.3389/fnetp.2022.809532 36187938 PMC9524168

[B28] Rogers J. (2004). Combined phase singularity and wavefront analysis for optical maps of ventricular fibrillation. IEEE Trans. Biomed. Eng. 51, 56–65. 10.1109/TBME.2003.820341 14723494

[B29] [Dataset] Schlemmer A. (2025). Wave tracker source code v0.2.0. 10.25625/TAG0PY

[B30] Seibertz F. Rubio T. Springer R. Popp F. Ritter M. Liutkute A. (2023). Atrial fibrillation-associated electrical remodelling in human induced pluripotent stem cell-derived atrial cardiomyocytes: a novel pathway for antiarrhythmic therapy development. Cardiovasc. Res. 119, 2623–2637. 10.1093/cvr/cvad143 37677054 PMC10730244

[B31] Steyer J. Lilienkamp T. Luther S. Parlitz U. (2023). The role of pulse timing in cardiac defibrillation. Front. Netw. Physiology 2, 1007585. 10.3389/fnetp.2022.1007585 36926106 PMC10013017

[B32] Suth D. Luther S. Lilienkamp T. (2024). Chaos control in cardiac dynamics: terminating chaotic states with local minima pacing. Front. Netw. Physiology 4, 1401661. 10.3389/fnetp.2024.1401661 39022296 PMC11252590

[B33] Voigt N. Li N. Wang Q. Wang W. Trafford A. W. Abu-Taha I. (2012). Enhanced sarcoplasmic reticulum Ca^2+^ leak and increased Na^+^- Ca^2+^ exchanger function underlie delayed afterdepolarizations in patients with chronic atrial fibrillation. Circulation 125, 2059–2070. 10.1161/CIRCULATIONAHA.111.067306 22456474 PMC4663993

[B34] Wilson G. Aruliah D. A. Brown C. T. Chue Hong N. P. Davis M. Guy R. T. (2014). Best practices for scientific computing. PLoS Biol. 12, e1001745. 10.1371/journal.pbio.1001745 24415924 PMC3886731

